# Prenatal parental involvement in decision for delivery room management at 22-26 weeks of gestation in France - The EPIPAGE-2 Cohort Study

**DOI:** 10.1371/journal.pone.0221859

**Published:** 2019-08-29

**Authors:** Cerise Levaillant, Laurence Caeymaex, Hélène Béhal, Monique Kaminski, Caroline Diguisto, Barthélémy Tosello, Elie Azria, Olivier Claris, Pierre Bétrémieux, Laurence Foix L’Hélias, Patrick Truffert, on behalf of the EPIPAGE 2 Extreme Prematurity Group

**Affiliations:** 1 CHU Lille Neonatal unit, EA Epidemiology and Quality of Care, Lille, France; 2 Department of Neonatology, Centre Hospitalier Intercommunal de Creteil, Créteil, France; 3 CEDITEC, University Paris Est Creteil, France; 4 Department of biostatistics, Univ. Lille, CHU Lille, Lille, France; 5 Inserm UMR, Obstetrical, Perinatal and Pediatric Epidemiology Research Team (Epopé), Center for Epidemiology and Statistics Sorbonne Paris Cité, DHU Risks in Pregnancy, Paris Descartes University, Paris, France; 6 Maternité Olympe de Gouges, Centre Hospitalier Regional Universitaire Tours, France; 7 Department of Neonatology, Assistance Publique-Hôpitaux de Marseille, Nord Hospital, Marseille, France; 8 Aix-Marseille University, CNRS, EFS, ADES, Marseille, France; 9 Maternity Unit, Groupe Hospitalier Paris Saint Joseph, Paris, France; 10 Department of Neonatology, Hospices Civils de Lyon, Hôpital Femme Mère Enfants; 11 Claude Bernard University, EAM, France; 12 Pôle Femme-Enfant, CHU, Rennes, France; 13 Sorbonne Université Paris, France, Service de Néonatologie, Hôpital Armand Trousseau, Assistance Publique-Hôpitaux de Paris, Paris, France; Centre Hospitalier Universitaire Vaudois, FRANCE

## Abstract

**Objective:**

Our main objective was to examine if parental prenatal preferences predict delivery-room management of extremely preterm periviable infants. The secondary objectives were to describe parental involvement and the content of prenatal counseling given to parents for this prenatal decision.

**Design:**

Prospective study of neonates liveborn between 22 and 26 weeks of gestation in France in 2011 among the neonates included in the EPIPAGE-2 study

**Setting:**

18 centers participating in the “Extreme Prematurity Group” substudy of the EPIPAGE-2 study.

**Patients:**

302 neonates liveborn between 22–26 weeks among which 113 with known parental preferences while parental preferences were unknown or unavailable for 186 and delivery room management was missing for 3.

**Results:**

Data on prenatal counseling and parental preferences were collected by a questionnaire completed by professionals who cared for the baby at birth; delivery room (DR) management, classified as stabilization or initiation of resuscitation (SIR) vs comfort care (CC). The 113 neonates studied had a mean (SD) gestational age of 24 (0.1) weeks. Parents of neonates in the CC group preferred SIR less frequently than those with neonates in the SIR group (16% vs 88%, *p* < .001). After multivariate analysis, preference for SIR was an independent factor associated with this management. Professionals qualified decisions as shared (81%), exclusively medical (16%) or parental (3%). Information was described as medical with no personal opinion (71%), complete (75%) and generally pessimistic (54%).

**Conclusion:**

Parental involvement in prenatal decision-making did not reach satisfying rates in the studied setting. When available, prenatal parental preference was a determining factor for DR management of extremely preterm neonates. Potential biases in the content of prenatal counselling given to parents need to be evaluated.

## Introduction

Extremely low gestational age neonates (between 22 and 26 weeks) account for 0.3% of live births in France [1]. Several studies have described their mortality and sequelae, showing that active perinatal care increases survival but is associated with neurodevelopmental disability among survivors [2–5]. These studies, describing outcomes for extremely preterm infants as a group, call into question any systematic policy of delivery room (DR) management at these gestational ages (GA). Each newborn’s individual long-term outcome and quality of life depends on perinatal characteristics and the postnatal family and social environment and thus remains impossible to predict before birth [6–8].

DR management for probable or imminent delivery at extremely low GA must therefore be discussed prenatally. In many countries, guidelines recommend that it be decided prenatally, on an individualized basis, and that the decision involve future parents after they receive clear information [9–12]. Nonetheless, in studies assessing predelivery counseling in this context, parents report a low level of involvement the decision-making process and a need for more personal involvement [13–18]. Mothers in Canada report that to make decisions they need more information about prematurity and about their roles and responsibilities in the care of their preterm baby [16]. Grobman et al showed that parents at three Chicago hospitals required clear and explicit information on management options and potential outcomes to understand the situation and be prepared to participate in the prenatal decision-making process [17]. Providing essential clear and detailed case-by-case information about outcomes is complex, for preterm birth initiates a long series of uncertainties [19]. Moreover, emotions play a role in this decision-making process during the periviable period—for both parents and physicians. Studies show that both the framing and communication of information determine the parents’ mental picture and thus influence their perception of the most appropriate decision [19,20].

Our principal objective was to determine whether prenatal parental preference for the management of their infants born at a GA of 22–26 completed weeks helps predict subsequent DR management in France. Prenatal preference was described by the healthcare providers who completed a questionnaire about delivery room management. As health-care professionals may influence parents, our secondary objective was to describe parental involvement in the decision-making process and to characterize the information the professionals provided, the tone of their presentation, and the degree of their agreement with parents in the prenatal decision.

## Patients and methods

### Study population

EPIPAGE-2 (Etude Epidémiologique sur les Petits Ages Gestationnels) is a population-based cohort of preterm children born at a GA of 22–34 completed weeks in France from May to December, 2011 [21]. This paper is based on a specific substudy of neonates born at 22–26 weeks, which was designed to explore the influence of parental preferences before delivery on the final decision about DR management, parental involvement in the predelivery decision-making process, and the characteristics (content and tone) of prenatal information given by the professionals. The study included all infants enrolled in the EPIPAGE-2 cohort born without severe malformations between 22 weeks^+0^ and 26 weeks^+6^ of gestation in one of the 18 maternity units (17 level-3 units of the 65 in the main study and one of the 214 level-2 units) that volunteered to participate. The 18 centers were located in 13 different regions in France.

### Ethics statement

Families received information and agreed to participate in the study before data collection began. The National Data Protection Authority (CNIL n°911009) and the appropriate committees, i.e., the Consultative Committee on the Treatment of Information on Personal Health Data for Research Purposes (No. 10.626) and the Committee for the Protection of People participating in Biomedical Research (No. CPP SC-2873), approved the study [21].

### Data collection

This study used two sets of data. The first came from the EPIPAGE-2 population cohort, collected as described elsewhere [21]. The second, designed for this specific study, comprised a questionnaire completed by the health-care providers involved in the predelivery counseling and another describing DR management, completed by the providers concerned. The primary outcome was DR management: either comfort care (CC) without any resuscitation maneuver, or stabilization or initiation of resuscitation (SIR) defined as administration of any respiratory support, from mask to intubation, including CPAP or any other form of resuscitation. The prenatal counseling, provided after a multidisciplinary medical meeting (i.e. generally involving an obstetrician, neonatologist, and midwife) to discuss the situation, was recorded in the mother’s chart.

As a family could have had several prenatal counsel sessions at different stages of the pregnancy, the total number of such sessions was noted as well as the parents present at each. Prenatal parental preference for CC or SIR, expressed at these meetings, was also collected; for families with multiple sessions, the final preference expressed was considered. Health-care providers also assessed the specific type of prenatal parental involvement: decision made without parental information (paternalism), parents received information but their preference was not requested (also paternalism), or decision considered the parents’ opinion (shared decision). Professionals also assessed the existence of disagreement between parents and themselves about the decision (and its temporary or definitive nature) and qualified the decision as medical or parental, on a Likert-type scale: “Finally, between a medical and a parental decision, where do you place this decision?” (from 1 = exclusively medical, to 5 = exclusively parental).

Providers also qualified the content of the information delivered to parents as: “medical: information on diagnostic, prognosis, and neonatal management” versus “medical with health care providers’ point of view, and/or personal values”; and also as “complete”, including all necessary information to decide versus “limited” (for various reasons: prognostic uncertainty, emergency situation, language barrier, limited parental comprehension, medical desire to protect the parents). The general tone of the information was described as stressing one of three options: “uncertainty”, “pessimism”, or “optimism”. Finally, the use of statistical data about outcome (yes/no), and the inclusion of the counseling content in the mother’s chart (yes/no) were noted.

Data from the entire EPIPAGE-2 cohort included infants’ characteristics: gestational age, birth weight, sex, and vital status after DR care. GA was expressed in completed weeks of gestation and defined as the best obstetric estimate, combining last menstrual period and ultrasound assessment. Maternal social and demographic characteristics (age, number of previous pregnancies, occupation and work status before pregnancy) were also recorded, as were data related to the current pregnancy (including but not limited to any fertility treatment, multiple pregnancy, antenatal corticosteroids (yes if > = 1 injection), and intrauterine transfer).

### Statistical analysis

We first compared neonates for whom prenatal parental preference was collected and available and those for whom it was not. We then compared neonatal characteristics and parental information for those receiving CC and those receiving SIR.

Categorical variables were described by frequencies and percentages, and continuous variables were described by means and standard deviation or medians and maximum/minimums. Comparative analysis was done using Chi-2 or Fisher test for qualitative variables, and Student test or analysis of variance tests for quantitative variables.

Fixed linear models were used at the univariate step because of the non-independence of newborns from multiple births. We first analyzed associations between perinatal characteristics, parental information variables, and DR management. Then, in the multivariate analysis to identify factors predictors of SIR, we considered the variables significant at a *P* value < .05 and with <10% of missing data. Birth weight was not retained because of its strong colinearity with GA. The statistical analysis used SAS 9.3 software.

## Results

### Study population

**Fig 1:**
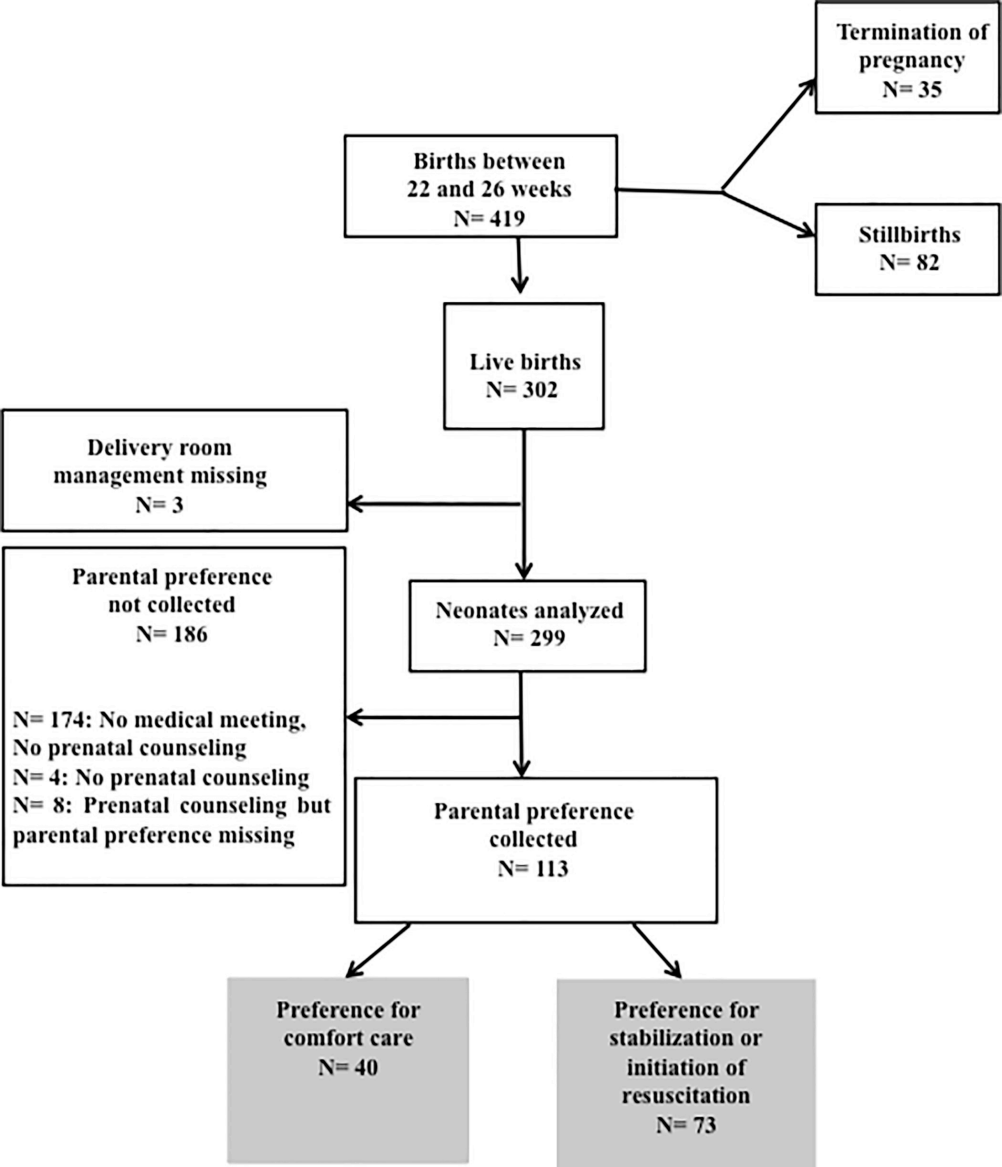
Flowchart of the study population.

Eighteen hospitals in 13 regions of France participated submitting 419 questionnaires, including 302 for neonates born alive at 22–26 completed weeks (Fig 1). Parents’ prenatally expressed preference was not available for 186 neonates, most often because parents’ preferences were not collected, either because no medical meeting had discussed planned DR management or, less frequently, because no parental counseling had taken place. Least often, information on prenatal parental preference was missing from the questionnaire.

Compared to those for whom parental preferences were known and available, these 186 neonates had a higher GA and birth weight and received antenatal corticosteroids more frequently (Table 1).

**Table 1:** Characteristics of infants and pregnancies according to whether a parental preference was collected prenatally.

	Prenatal parental preference not collected	Prenatal parental preference collected	p
	n = 186	n = 113	
**Infants**			
GA mean (SD)	25.2 (1)	24.4 (1)	< .001
Birth weight mean (SD)	769 (153)	693 (130)	< .001
Sex male n (%)	102 (55)	67 (59)	0.58
**Status n (%)**			< .001
Death in delivery room	28 (15)	45 (40)	
Death in NICU	47 (25)	25 (22)	
Alive at discharge	111 (60)	43 (38)	
**Pregnancies**			
Fertility treatment n (%)	45 (25)	19 (17)	0.17
Antenatal corticosteroids n (%)	155 (85)	72 (65)	0.002

SD: standard deviation

Prenatal parental preference was known and available for more than half the infants born at 24 weeks or less. Primary DR CC was given to 10% of the neonates for whom we lacked prenatal parental preferences (Table 2).

**Table 2:** Delivery room management according to gestational age and existence of prenatal parental preferences for delivery room management.

	Prenatal parental preference for DR management	
	Not collected	Collected
	n = 186	n = 113
**Comfort care (CC) n (%)**	19 (10%)	37 (33%)
**Gestational age, completed weeks n (%)**		
**22 weeks**		
CC n (%)	2/2 (100)	4/4 (0)
SIR n (%)	0/0 (0)	0/0 (0)
**23 weeks**		
CC n (%)	8/8 (100)	15/17 (88)
SIR n (%)	0/8 (0)	2/17 (12)
**24 weeks**		
CC n (%)	8/30 (27)	13/36 (36)
SIR n (%)	22/30 (73)	23/36 (64)
**25–26 weeks**		
CC n (%)	1/146 (1)	5/56 (9)
SIR n (%)	145/146 (99)	51/56 (91)

CC: comfort care; SIR: stabilization or initiation of resuscitation; DR: delivery room

### DR management according to prenatal parental preference

Among the 113 newborns with data about prenatal parental preference, 37 (33%) received CC and 76 (67%) SIR. For 98 infants (87%), DR management matched the prenatally expressed parental preference; of the remaining 15 (13%), 6 received CC although their parents had chosen SIR (1 at 22 weeks, 3 at 23 weeks, 2 at 24 weeks), and 9 received SIR despite their parents’ preference for CC (3 at 24 weeks, 5 at 25 weeks and 1 at 26 weeks).

Table 3 shows the characteristics of neonates, mothers, and pregnancies according to DR management. Gestational age and birth weight were lower in the CC group and antenatal corticosteroids less frequent. Parents in the CC group had preferred SIR prenatally significantly less frequently than in the SIR group (16% vs 88%, *p <* .*001)*. Emergency delivery explained more than half of our cases of limited information; defined by direct admission to the DR, it was slightly (although not statistically significant) less frequent in the SIR group.

**Table 3:** Characteristics of newborns, mothers, pregnancy, and urgency of delivery according to type of delivery room care.

	Comfort Care	SIR	p
	n = 37	n = 76	
**Newborns**			
Gestational age (GA) mean (SD)	23.5 (0.93)	24.9 (0.8)	< .001
GA in weeks n (%):			< .001
*22 weeks*	4 (11)	0	
*23 weeks*	15 (40)	2 (3)	
*24 weeks*	13 (35)	23 (30)	
*25–26 weeks*	5 (14)	51 (67)	
Birth weight m (SD)	627 (131)	724 (119)	.007
Sex, male n (%)	21 (57)	46 (60)	.77
1-min Apgar score, med (Q1; Q3)*	1 (1; 1)	4 (2; 6)	.03
Died in delivery room n (%)	36 (97)	9 (12)	< .001
**Mothers**			
Age m (SD)	31.1 (6.6)	29.3 (5.6)	.29
Number of previous pregnancies md (Q1; Q3)	1 (0; 1)	0 (0; 1)	.13
Country of birth, France n (%)*	17 (60.7)	48 (75.0)	.30
Occupational activity n (%)	14 (43.8)	48 (65.8)	.09
**Pregnancy**			
Fertility treatment n (%)	3 (8.3)	16 (21.3)	.18
Multiple pregnancy n (%)	17 (46.0)	25 (32.9)	.27
Antenatal corticosteroids n (%)	9 (25.0)	63 (84.0)	< .001
Intrauterine transfer n (%)	18 (46.7)	50 (65.8)	.13
**Factors suggesting emergency**			
Direct admission to DR n (%)	12 (33)	11 (15)	.08

n: number; SD: standard deviation; md (Q1; Q3): median (first and third quartiles); CC: comfort care; SIR: stabilization or initiation of resuscitation; DR: delivery room

* variable with more than 10% missing data.

### Professionals’ characterization of parental involvement in the decision-making process for DR management

Table 4 shows the specific health-care providers and parents present at predelivery counseling sessions; the mean number of parents’ meeting was 1.85.

**Table 4:** Participants in predelivery counseling about delivery room management, reported by health-care professionals.

N with available information	Total	CC	SIR	p
	n/N (%)	n /N (%)	n/N (%)	
**Multidisciplinary medical meeting before parental counseling**	77/110 (70)	29/36 (81)	48/74 (65)	.19
**Professional**				
Pediatrician alone n (%)	93/112 (83)	26/36 (72)	67/76 (88)	.08
Obstetrician alone n (%)	68/111 (61)	32/36 (89)	36/75 (48)	.003
Pediatrician and obstetrician n (%)	18/112 (16)	7/36 (19)	11/76 (14)	.52
**Mother living with partner (N = 98)**				
Parents present at each n (%)	70/96 (73)	19/28 (68)	51/68 (75)	.02

DR: delivery room; CC: comfort care; SIR: stabilization or initiation of resuscitation. Denominators vary according to the number of missing data.

Professionals characterized parental involvement in the prenatal decision-making process as “Information with documentation of parental wishes” in 54 cases (48%) - 14 (38%) in the CC group and 40 (53%) in the SIR group; “Information with documentation of a parental agreement” in 51 cases (45%)– 19 (51%) in the CC group and 32 (42%) in the SIR group; and “Parental information without documentation of parental preference” in 7 cases (6%).

The results from the Likert-type scale showed an exclusively medical decision or directive counseling (score of 1) for 16% (15/91), a shared decision (score from 2 to 4) for 81% (73/91), and an exclusively parental decision (score of 5) for 3% (3/91). Mean and standard deviation (SD) Likert-type scale results were 2,7 (0,95) with no difference between CC and SIR groups (p .64).

Finally, 6 cases of disagreement between professionals and parents were reported, 3 temporary and 3 definitive (2 infants born at 24 weeks, 3 at 25 weeks, and 1 at 26 weeks): 3 parents were opposed to CC and 3 to SIR.

Table 5 summarizes the professionals’ descriptions of information provided. Reasons for limited information were emergency delivery (15/28), parental failure to understand the situation (6/28), and a language barrier (4/28).

**Table 5:** Characteristics of information provided at predelivery counseling, reported by health-care professionals.

N with available information	Total	CC	SIR	p
	N = 112	n/N (%)	n/N (%)	
**Content of information delivered to the parents**				.69
Medical*, no personal viewpoint n/N (%)	80/112 (71)	27/36 (75)	53/76 (70)	
Medical* and personal viewpoint** n/N (%)	32/112 (29)	9/36 (25)	23/76 (30)	
**Volume of the information delivered**				.21
Complete, including all information necessary for decision n/N (%)	83/111 (75)	31/37 (84)	52/74 (70)	
Limited n/N (%)	28/111 (25)	6/37 (16)	22/74 (30)	
**Principal message you wanted to give to the parents?**				< .001
Uncertainty n/N (%)	46/110 (42)	2/37 (5)	44/73 (60)	
Pessimism n/N (%)	59/110 (54)	33/37 (90)	26/73 (36)	
Optimism n/N (%)	5/110 (5)	2/37 (5)	3/73 (4)	
**Did you mention statistical data: yes n/N (%)**	33/107 (31)	6/35 (17)	27/72 (37)	.08
**Counseling content reported in mother's chart n/N(%)**	88 /107 (82)	35/37 (95)	53/70 (76)	.06

*Medical, defined as diagnostic, prognostic, and modalities of care

**Personal viewpoint, opinion and personal values of the health-care professional.

CC: comfort care; SIR: stabilization or initiation of resuscitation. Denominators vary according to the number of missing data.

### Factors associated with SUR in the delivery room: Multivariate analysis

A Multivariate analysis of factors associated with SIR in delivery room, with odds ratios adjusted for all variables in the model, showed that prenatal parental preference for SIR increased the probability of DR SIR management (OR = 61.2 [5.2; 713.5]; *p* < .001). Higher GA (ORa 7.8 [1.5; 39.7]; *p* .02) and antenatal corticosteroid exposure (ORa 8.9 [1.0; 84.4]; *p* .05) were also predictive factors of SIR. Counselors’ professional (Obstetrician) was not predictive of SIR (ORa 0.2 [0.02; 2.7]; p .23).

## Discussion

This is the first study to prospectively assess the influence and concordance of parents’ prenatally collected preference with actual DR management for extremely preterm infants. It is also the first to explore professionals’ real-life information practices and parental involvement in prenatal counseling and the decision-making process. Our study shows that prenatal parental preference was a determining factor in the decision about DR management for a cohort of neonates born at 22–26 weeks in France. Professionals described shared decision-making and alignment between parental preference and DR management for those whose prenatal preference was known. This result is consistent with French and international guidelines about the management of extremely preterm births [9–12, 18].

But this study also shows that prenatal parental preference was unknown or unavailable for more than half the patients in our cohort. Of interest, two-thirds of the neonates in this subgroup were born after 24 weeks GA, and all but one of these received SIR without any parental preference expressed in most cases without a multidisciplinary meeting; those aged 22 and 23 weeks received CC in the absence of an available parental preference. This was consistent with practices in France and in other countries in 2011 [20–22]. At 24 weeks, however, the decision should not only be based on GA but include other prognostic factors such as birth weight and antenatal corticosteroid administration. Parents’ prenatal preference should also play an important role.

The recommendations, while partially based on GA, encourage professionals to use an individualized approach that includes other factors [6]. In our study, the type of care was associated with GA [23], but prenatal parental preference remained the main decisional factor after adjustment for GA. Guidelines [9–12] are organized by gestational age subgroup, although models relying only on GA are not reliable for predicting outcomes [6]. Other prognostic factors have thus been suggested for inclusion in the decision-making process, although not to predict individual outcome. French guidelines are currently being modified with an emphasis on parental involvement.

The difference in the antenatal corticosteroid administration rates between the CC and SIR groups might reflect a prenatal decision for CC among the lowest-GA newborns. Nonetheless, this treatment is simultaneously a prognostic factor [24,25], a possible consequence of a prenatal decision for SIR, and a decisional factor in DR management of periviable infants [6] and should therefore be administered in any case if clinically indicated, rather than being used as an argument in the decision.

Contrary to guidelines, obstetricians and pediatricians jointly provided parental counseling in fewer than 20% of our cases [11,12,26]. Obstetricians provided counseling more often in the CC than in the SIR group. Studies have shown that obstetricians overestimate poor prognosis [27] and differ from pediatricians in their prenatal DR decisions [28], and that joint prenatal counseling generates a more consensual process [26]. Parents have also reported their need for consistent messages in these situations [29]. In one fourth of our cases, only the mother received counseling. This practice appears inappropriate, as the father’s presence should result in better expression of the family choice, decreased inhibition, and comfort for the mother [30–32].

Professionals most often described the information as complete and without inclusion of their personal opinion or values. These results are in accordance with the guidelines recommending objective transparent information and with parental opinions about what prenatal counseling should be [15,16,32]. Nonetheless, what the professional perceives as complete (all the information needed to make a decision) may not always meet parental criteria of completeness: parents’ needs vary, and the ideal content of prenatal counseling is not the same for every parent. Moreover, many parents do not use all the information given [33]: as in other medical settings, patients consider only a part of the information they receive in making decisions about care [34]. Emergency situations and language barriers can each hinder parental autonomy. The negative corollary of the emergency situation must be limited by organizing the first meeting with the parents as quickly as possible [17], while interpreters must be provided to families with language barriers, especially as cultural differences may also complicate their communication with health-care providers. Statistical data, rarely mentioned by the professionals in our study, can be useful if they are relevant and understandable; that is, if they reflect national data and if their framing does not lead to bias or misunderstanding [19,35]. Nonetheless, because French statistics are based on results from practices that follow French recommendations, they may create a self-fulfilling prophecy [19]. In practice, physicians should know the important international epidemiological data but use them with caution, because they are generally difficult for families to interpret [35].

Charles et al [36] describe an “informed decision-making model” in which doctors provide complete medical information without including their personal point of view and a “shared decision-making model” that is not exclusively limited to medical considerations. Professionals in our study appear to use both models.

Parents participate in the decision-making process for their baby. Yet our study also shows that the tone or connotation of the information probably influences the prenatal parental preference (90% pessimistic in the CC group and 63% uncertain in the SIR group). The influence of a pessimistic approach might be greater in non-religious families [37], but globally, emotions always influence decisions [38]. Optimistic messages were rarely reported in our study. Health-care providers are known to be reluctant to encourage false hope [17]; existing French data and practice suggest that professionals in France would prefer to underline uncertainty or pessimism rather than express the possibility of a good prognosis. They probably understood pessimism to mean the presentation of the negative perspective, without balancing it with the positive possibilities. Parents say, however, that they want doctors to share all the information and not just the “gloomy stuff”, especially when the mother is still pregnant and parents need hope [17]. Haward et al showed that the presentation of information in positive form positively influenced parental decision-making [39].

Professionals qualified the decision as shared in most cases, consistent with studies showing families’ desire to share the decision with the medical team [33,40]. Parents prefer decisions individualized to their personal and familial situation [14–16,19,41]. We found that after prenatal counseling the parents and medical team disagreed for fewer than 20% of the neonates.

This study has three main limitations. The most prominent is that prenatal parental preference for DR management was either unknown (not collected) or unavailable for more than 50% of the cases. Most of these neonates were born at 25 or 26 weeks, and 99% of this subgroup received SIR; the subgroup with a GA of 22–23 weeks was a minority, and all of them received CC. This suggests that before birth, at these GA, physicians did not involve parents in a discussion and applied SIR and CC following the national recommendations without taking into account their opinion. Even at 24 weeks, parents’ preferences were not always known, either because no one asked them their preferences or because these were not reported in the hospital records. A second limitation is the potential influence of a bias in the tone of information given to the parents, which was negative or neutral in the majority of cases despite consistently better results both in survival and in neurodevelopmental outcomes have been reported in studies in different countries. This is difficult to explain but we might hypothesize that ignorance of the results of these studies or self-fulfilled prophetic « poor » outcome in local settings might influence professionals’ idea of outcome. Finally, another limitation is the possible bias due to the self-interpretation by the health-care providers of the role given to parents in the decision-making process. Health-care providers might have reported shared decision-making in order to be in agreement with recommendations while parents’ perceptions or reports might have been different [41].

We assessed the tone of prenatal counseling to identify such possible bias in current communication practices. Uncertainty seems the most accurate message to convey, and the possibility of both positive and negative outcomes should be stated according to up-to-date data on outcomes. In recent years, prenatal counseling has been viewed as a dialogue, a conversation, rather than as the doctor’s monologue [29], and the importance of interaction has been stressed. Parents need the opportunity to express their personal views, and doctors must adjust the content of their information to parents' priorities. Authors working on risk communication report that effective communication requires doctors to strive to show both their competence and their caring [12,16,17,33,35], for it is these qualities together that help to build trust [35].

Our study nonetheless shows that French physicians still express their opinion in prenatal interviews, and, moreover, with a mainly uncertain or pessimistic prognosis. This appears to be in contradiction with their view considering themselves to give “complete information without inclusion of their personal opinion or values”.

The best attitude is still being debated because of the concern that full autonomy may attribute too much responsibility to families and thus assign them too much guilt. Some authors thus consider that a middle course is the best alternative [42]. An individualized approach is necessary.

## Conclusion

In 2011, in France, parental involvement in prenatal decision-making did not reach satisfying rates in the studied setting. When available, prenatal parental preference was a determining factor for DR management of extremely preterm neonates. Potential biases in the content of prenatal counselling given to parents need to be evaluated. An evolution of practices based on updated guidelines is therefore necessary including joint parental counselling by paediatrician and obstetrician in presence of both parents, and for professionals, a more accurate knowledge of national and international data.
